# The Prognosis in Palliative care Study II (PiPS2): study protocol for a multi-centre, prospective, observational, cohort study

**DOI:** 10.1186/s12904-018-0352-y

**Published:** 2018-08-13

**Authors:** Anastasia K. Kalpakidou, Chris Todd, Vaughan Keeley, Jane Griffiths, Karen Spencer, Victoria Vickerstaff, Rumana Z. Omar, Patrick Stone

**Affiliations:** 1 Marie Curie Palliative Care Research Department, Division of Psychiatry, UCL, 6th Floor, Maple House, 149 Tottenham Court Road, London, W1T 7NF UK; 2 The School of Nursing, Midwifery and Social Work, University of Manchester, Manchester, M13 9PL UK; 3 Derby Teaching Hospitals NHS Foundation Trust, Derby, DE1 2QY UK; 4 Department of Statistical Science, UCL, London, WC1E 7HB UK

**Keywords:** Cancer, Palliative care, Prognosis, Observational study

## Abstract

**Background:**

More accurate methods of prognostication are likely to lead to improvements in the quality of care of patients approaching the ends of their lives. The Prognosis in Palliative care Scales (PiPS) are prognostic models of survival. The scores are calculated using simple clinical data and observations. There are two separate PiPS models; PiPS-A for patients without blood test results and PiPS-B for patients with blood test results. Both models predict whether a patient is likely to live for “days”, “weeks” or “months” and have been shown to perform as well as clinicians’ estimates of survival. PiPS-B has also been found to be significantly better than doctors’ estimates of survival. We report here a protocol for the validation of PiPS and for the evaluation of the accuracy of other prognostic tools in a new, larger cohort of patients with advanced cancer.

**Methods:**

This is a national, multi-centre, prospective, observational cohort study, aiming to recruit 1778 patients via palliative care services across England and Wales. Eligible patients have advanced, incurable cancer and have recently been referred to palliative care services. Patients with or without capacity are included in the study.

The primary outcome is the accuracy of PiPS predictions and the difference in accuracy between these predictions and the clinicians’ estimates of survival; with PiPS-B being the main model of interest. The secondary outcomes include the accuracy of predictions by the Palliative Prognostic Index (PPI), Palliative Performance Scale (PPS), Palliative Prognostic score (PaP) and the Feliu Prognostic Nomogram (FPN) compared with actual patient survival and clinicians’ estimates of survival.

A nested qualitative sub-study using face-to-face interviews with patients, carers and clinicians is also being undertaken to assess the acceptability of the prognostic models and to identify barriers and facilitators to clinical use.

**Discussion:**

The study closed to recruitment at the end of April 2018 having exceeded the required sample size of 1778 patients. The qualitative sub-study is nearing completion. This demonstrates the feasibility of recruiting large numbers of participants to a prospective palliative care study.

**Trial registration:**

ISRCTN13688211 (registration date: 28/06/2016).

## Background

Patients approaching the ends of their lives, their relatives and clinicians all value accurate prognostic information [1–5]. This information is routinely provided by clinicians using their clinical intuition. However, clinicians’ estimates of survival (CES) are often inaccurate and over-optimistic [6]. The need for more accurate methods of prognostication was highlighted by the Neuberger report [7] into the short-comings of the implementation of the Liverpool Care Pathway [8].

Determining more accurately how long patients with advanced cancer have left to live would enable both patients and their relatives to make plans for their future [3]. It would also aid clinicians to target treatments to those patients most likely to benefit and it would safeguard other patients from receiving treatments that they are unlikely to benefit from [9]. Prognostic information may help clinicians to plan services and to ensure that patients are cared for in the most appropriate environment. More reliable prognostic estimates may also facilitate the identification of patients for inclusion on palliative care registers [10] and the prioritisation of patients who are referred to palliative care services.

The Prognosis in Palliative care Scales (PiPS) are predictive models of survival and were previously developed by members of our research team in order to provide an objective aid to clinicians’ intuition [9]. The original study recruited prospectively a cohort of 1018 patients with advanced cancer, who were no longer undergoing disease-modifying treatment. This was a multi-centre study involving 18 specialist palliative care services across England. Separate prognostic models were created for patients without (PiPS-A) or with (PiPS-B) available blood test results. The PiPS scores were able to predict whether a patient was likely to live for “days” (less than 14 days), “weeks” (between 2 and 7 weeks) or “months” (2 months or longer). These categories were chosen as they seemed to have the greatest face validity among clinicians. Both PiPS models were shown to perform as well as CES. The PiPS-B prognostic estimate was found to be significantly better than doctors’ prognostic estimates. Before recommending PiPS for use in routine clinical practice, it is important to check that the models provide accurate and reliable estimates of survival in a new group of patients.

More recently, two research studies have published validation data about the PiPS models [11, 12] in different clinical settings. Baba and colleagues (2015) [11] undertook an independent validation of the PiPS models in Japanese cancer patients and reported that the PiPS instrument performed as well as in Gwilliam’s original study [9]. However, the Japanese study did not compare the accuracy of PiPS to the accuracy of CES. Differences in cancer epidemiology and oncology practice between Japan and UK may also be a relevant consideration. The study by Kim and co-workers (2014) [12] reported that the PiPS instruments performed approximately as well as in the original study. However, the Kim study included a relatively small number of participants and the study population was restricted to palliative cancer patients in a specialist cancer hospital in South Korea. No large scale validation has yet been undertaken in the UK.

Taken together, these two studies strengthen the case for undertaking a large-scale validation study in the UK using CES as a comparator. Based on systematic reviews [13, 14], four other prognostic models have been identified that might also be useful in clinical practice and which are in need of further evaluation. These are the Palliative Prognostic Index (PPI) [15], the Palliative Performance Scale (PPS) [16], the Palliative Prognostic (PaP) [17] score and the Feliu Prognostic Nomogram (FPN) [18].

The PPI and the PPS can both be calculated without the need for a blood test (like PiPS-A). The PPI model stratifies patients into three groups; survival shorter than 3 weeks; shorter than 6 weeks; or more than 6 weeks [15]. The PPI has shown a high accuracy level in patients with short estimates of survival [19]. The PPS is a measure of functional status and is one of the variables included in the PPI score. Although not specifically designed as a prognostic instrument, and therefore lacking some face validity as a stand-alone prognostic tool, the PPS has been found to have prognostic significance in patients with advanced disease [20, 21]. Using data from large observational studies, the PPS can be used to predict the probability of dying across a range of survival times [20].

The PaP and the FPN, both require blood test results (like PiPS-B). The PaP score classifies patients into three risk groups based on a 30-day survival probability of less than 30%; between 30 and 70%; and more than 70% [17]. There is increasing evidence to support its validity in a variety of settings [22–26]. The total PaP score was shown in one study to be more accurate than a simple clinician prediction of survival [27]. One practical concern with the PaP score is that it relies on CES. This can make the PaP challenging to use when clinicians are unsure about survival times or when an “objective” estimate is required that is free from the influence of CES. The FPN predicts survival at 15, 30 and 60 days [18]. In one study the FPN was found to be more accurate than the PaP [18] and does not rely on subjective CES.

The PiPS2 study was designed with the overall aim of validating the accuracy of the PiPS-A and PiPS-B [9]. The primary objectives of the study are: to validate the PiPS models and; to compare the performance of PiPS-B against CES. The secondary objectives are to validate other selected prognostic models (PPI, PPS, PaP and FPN).

A nested qualitative sub-study has also been embedded in the PiPS2 study to assess the acceptability of the prognostic models to patients, carers and clinicians and to identify barriers and facilitators to clinical use. This is particularly relevant because in clinical practice it is often the relatives and carers of non-competent or semi-conscious patients who most wish to have access to accurate prognostic information.

## Methods

### Study design

This is a multi-centre, prospective, observational, cohort study to validate various prognostic models in patients with advanced, incurable cancer. The study involves patients from 28 palliative care services across England and UK and is sponsored by University College London.

Patients were recruited in three palliative care settings:

- community palliative care teams (including day hospice and palliative care outpatients)
- hospital palliative care teams
- inpatient palliative care units

The planned recruitment period for the validation study was from August 2016 to the end in April 2018. At least three months after all recruitment has ended, a list of study participants will be sent to the Health and Social Care Information Centre (HSCIC) in order to determine dates of death. The accuracy of the studied prognostic models will then be compared against actual survival.

Following Medical Research Council Guidance [28] a subset of patients who agreed to participate in the main study, their next-of-kin (informal carers), patients who declined to participate in the main study, and health care professionals, were recruited into a nested qualitative sub-study to explore views about prognostication and the use of prognostic tools.

### Participants

The study involved both patients with or without capacity to consent to participate. Many patients at the end of their lives become confused, semi-conscious, and comatose or may have pre-existing cognitive impairment, they therefore frequently lack capacity to consent to participate in research. The inclusion of patients who lack capacity is therefore of great importance as the study population should be representative of those patients commonly seen in palliative care services. Capacity was assessed by the attending clinician using the Department of Health guidance [29]. If capacity was in doubt, the clinician carried out a capacity test as provided by the Royal College of General Practitioners’ Mental Capacity Act Toolkit for Adults in England and Wales 2011 [30].

#### Inclusion criteria

- Patients with locally advanced or metastatic incurable cancer. A patient with incurable cancer has an estimated prognosis of survival of less than one year.
- Men and women aged 18 years or over.
- Patients who had recently been referred to palliative care services. Recent referral has various meanings across different palliative care settings. For a community, day hospice or palliative care outpatient, “recent” means that the patient should have had fewer than three previous contacts with the palliative care service before they are recruited to the study. For inpatient palliative care patients (including hospital support teams), “recent” referral means that the patient should have been first seen by a member of the palliative care team no more than 7-days previously. The date of referral was considered the date the patient had first been seen by a member of the relevant palliative care team.
- Sufficient English language skills for patients with capacity to read and understand the Patient Information Sheet and undertake study assessments.

#### Exclusion criterion

Patients receiving (or planned to receive) treatment with curative intent at the time of consent. Patients receiving palliative treatment were still eligible to participate.

### Study assessments

Data were collected at a single time point and were usually obtained from a review of the medical notes or from a discussion with clinical staff. If patients were able to respond to questions data might have partly been obtained directly from them. The data that were required for the calculation of each prognostic score are shown in Table 1.

**Table 1:** Data required for the calculation of prognostic models

Variable	Prognostic model					
	PiPS-A	PiPS-B	PPI	PPS^a^	PaP	FPN
ECOG performance status	X	X				X
General health status	X	X				
Abbreviated Mental Test Score > 3	X	X				
Primary breast cancer	X					
Primary prostate cancer	X	X				
Distant metastases (any)	X	X				
Liver metastases	X					
Bone metastases	X	X				
Anorexia	X	X			X	
Dysphagia	X					
Dyspnoea	X		X		X	
Weight loss in last month	X					
Pulse rate	X	X				
Fatigue		X				
PPS^a^ score			X	X		
Oral intake			X			
Oedema			X			
Delirium			X			
CES					X	
KPS					X	
Time to terminal disease^b^						X
Blood Results:						
White blood count		X			X	
Lymphocyte count		X			X	X
Neutrophil count		X				
Platelet count		X				
Urea		X				
Albumin		X				X
Alkaline phosphatase		X				
Alanine transaminase		X				
C-Reactive protein		X				
Lactate dehydrogenase						X

^a^Despite PPS being designed as a measure of functional status, it does have prognostic significance and was therefore included in the prognostic models this study intends to validate. PPS is listed in both “Variable” and “Prognostic Model” sections as it is one of the variables used for the calculation of the PPI score ^b^Time to terminal disease refers to the time between the initial cancer diagnosis and the date at which the cancer became incurable (i.e., time at which deemed to be inoperable or became metastatic)

#### Demographic, disease and treatment related data and capacity recording

Demographic details of enrolled patients, such as age, gender and current patients’ location (e.g., home, hospital, hospice) were recorded. National Health Service (NHS) number and date of birth were also recorded to be sent to the HSCIC. Information on the nature and site of primary tumour and sites of metastases (if any) were also collected. Additionally, patients’ capacity to consent to participate in the study was documented.

#### Key symptoms

The presence or the absence of key symptoms was recorded (this was required for the scoring of the prognostic models); anorexia, dysphagia, dyspnoea, fatigue and weight loss.

#### Abbreviated mental health score

This is a test that assesses cognitive function [31]. This test is not applicable to patients without capacity, who were attributed scores of zero.

#### Clinical assessments

Clinical assessments included:

1. Recording of the presence or the absence of ascites, peripheral oedema, delirium and decreased oral intake. Pulse rate was also measured over one minute.
2. The Eastern Co-operative Oncology Group (ECOG) performance status [32]. This is a measurement of patients’ level of everyday functioning.
3. A 7-point scale of observer-rated health status. The scores range from very poor (point 1) to excellent (point 7).
4. The Karnofsky Performance Scale (KPS) [33] this is used to assess patients’ functional impairment.
5. The PPS [16]. This is one of the prognostic models that this study aims to validate. It describes the patients’ current ambulatory level; activity level and extent of disease; self-care abilities; intake and conscious level.
6. Estimation of the time to terminal disease by the clinicians. This is defined as the time elapsed between the diagnosis and development of incurable disease and is required to calculate the FPN.

#### Blood test results

For patients with capacity a fresh blood specimen must have been taken. For patients without capacity there was no requirement to take a fresh blood specimen, however if a blood sample was being taken for another reason as a part of routine clinical care within 72 h of study enrolment then the relevant tests were requested. Even if no new specimen was being taken from a patient without capacity, if relevant results were available within 72 h of being enrolled in the study then these results were recorded.

The blood test results required are: white blood count, lymphocyte count, neutrophil count, platelet count, urea, albumin, alkaline phosphatase, alanine transaminase, C-reactive protein, lactate dehydrogenase. Blood specimens were processed locally in the routine clinical laboratory using usual arrangements.

#### Clinicians’ estimates of survival

The attending doctor and nurse estimated survival of study participants independently. When the estimates agreed then this represented the combined multi-professional prediction. When they were discordant, the doctor and nurse discussed the case and reached a consensus. In order to characterise the prognosticators in more detail they were asked to provide information about themselves (i.e., age, gender, professional training and years of specialist experience). Clinicians were also asked to provide their prognostic estimates using a number of different formats in order to facilitate comparison with the outputs of the prognostic scores. Clinicians were asked: to provide approximate estimates of length of survival - “days” (0–13 days); “weeks” (2–7 weeks); “months+” (2 months or longer); to provide more specific estimates of survival to the nearest week (from < 1 week to > 12 weeks); to estimate the probability of survival at specific time points (1 day; 3 days; 7 days; 15 days; 30 days and 60 days).

### Data management

A study database for the storage, management and analyses of identifiable data has been developed via a secure web application named “REDCap” (Research Electronic Data Capture) and using the UCL Data Safe Haven secure system. This system has been certified to the ISO27001 information security standard and conforms to the NHS Information Governance Toolkit (http://www.ucl.ac.uk/isd/itforslms/services/handling-sens-data/tech-soln).

The rest of the (non-identifiable) study data have been sent to a separate database (i.e., electronic Case Report Form - eCRF) that has been created and supported by a company called “Sealed Envelope” (https://www.sealedenvelope.com/). Data from the paper CRFs have manually been transferred to the eCRFs by the researchers at each participating site.

A data monitoring plan has also been put in place to ensure optimal data quality across sites.

### Recruitment procedure

#### Patients’ identification

In each participating service, members of the clinical team maintained a screening log of all new referrals to the service. For the patients who were not eligible to participate, the screening log records the following information: age range, gender, reason for ineligibility.

#### Consent procedure

Study procedures differed between patients with and without capacity.

##### Patients with capacity

Eligible patients were approached by a member of the clinical team about participation in the study. If eligible patients were not approached by a team member then the reason for failure to do so was recorded on the screening log.

Potential participants who had been approached by a member of the clinical team were asked if they are be willing to speak to a member of the research team and are handed a Patient Information Sheet (PIS). A member of the research team then discussed the study with the patient and sought their consent to participate. For community patients this discussion might have occurred over the telephone. Written informed consent was usually obtained at least 24 h after the PIS had been handed out but could have been obtained on the same day if it was more convenient and acceptable for the patient to do so. If the patient declined to participate in the study then the reason for this (if known) was documented on the screening log.

##### Patients without capacity

For patients without capacity a personal consultee was sought for advice. For patients with no personal consultee, the advice of a nominated consultee was sought. The nominated consultee was usually another doctor working in the hospital/hospice (who was not involved in the research), a social worker, a chaplain or the patient’s General Practitioner (GP).

In a similar manner to the approach adopted for patients with capacity, consultees of patients without capacity were advised that it was usual practice to wait for 24 h before giving assent. If the consultee gave telephone advice for the patient to be included in the study but they are unable to visit the unit to provide written evidence of assent then verbal agreement was initially deemed to be sufficient to allow the research team to enrol the patient in the study and start data collection. However, in these circumstances an assent form was posted to the consultee to be signed and returned to the research team within two weeks of the patient being enrolled in the study. If no signed assent form was received then the patient was withdrawn from the study and all data were destroyed.

##### Patients with fluctuating capacity

Patients who temporarily lacked and then recovered capacity were informed about their involvement in the study and have the opportunity to withdraw or confirm participation [9].

### Outcome measures

#### Primary outcome measures

The survival of patients (measured from the date patients consented to participate), the CES and the predictions of the PiPS-A and the PiPS-B prognostic models.

#### Secondary outcome measures

The predictions produced by the PPI, PPS, PaP and FPN.

### Sample size

The sample size calculations are based on data collected during the original study [9].

#### For the comparison between PiPS-B model predictions and CES

PiPS-B is the primary model of interest for this research. To show at least a 5% improvement in correct predictions using PiPS-B compared to clinicians’ predictions, assuming 80% power and 5% significance level and using a McNemar’s test, a total of 1267 patients will be required. The formula and the software used for these calculations are based on the work produced by Machin and co-workers (2009) [34]. It is estimated that in order to obtain 1267 complete data sets it is be necessary to recruit 1334 patients with capacity (assuming 5% missing data). In order to recruit 1334 patients with capacity it is be necessary to recruit 1778 patients in total (assuming 25% of patients will lack capacity).

#### For the validation of the PiPS models

To validate predictions from a risk model it has been recommended that the validation data should have at least 100 events [35]. The validation data for the proposed study will involve several centres. There is no guidance on sample size calculation for multi-centre validation data. We expect clustering of patients within centres to be minimal based on other studies in community care [36]. However, to be conservative we have inflated the number of events required in the validation data to 150. Assuming an event rate of 17.8%, based on the original study, we will require 843 patients to validate the PiPS-B model. In fact, 1778 patients will be recruited, most of who will be able to provide data for the validation of both PiPS-A and PiPS-B, thus this sample size will be more than adequate to validate both models. Using similar arguments, the proposed sample size will also be more than sufficient to simply validate the other prognostic models (i.e., PPI, PPS, PaP and FPN).

### Planned analyses

#### Descriptive analysis

Predictors and outcomes will be summarised using descriptive analysis. Categorical predictors will be reported as raw numbers and percentages. Continuous variables will be summarised using mean or median and standard deviation or interquartile (IQ) range as appropriate. The percentage of values missing for each predictor will also be presented. The survival times of patients will be summarised using median and IQ ranges, and Kaplan Meier graphs.

#### Primary analyses

##### Validation of PiPS models (comparison between PiPS model predictions and actual patients’ survival)

The PiPS models will be validated as they were presented for use in the original study by Gwilliam and co-workers [9]. For both PiPS-A and PiPS-B, two separate models have been developed to predict the two week (14 day) and two month (56 day) survival of patients, thus, generating three prognostic categories i.e., less than two weeks, two weeks to two months and greater than two months.

The discriminatory ability of the models will be assessed using the C-statistic. Separate C-statistics will be calculated for the “two weeks” and the “two months” models. The PiPS online calculator provides (see www.pips.sgul.ac.uk) a prediction as to whether a patient will survive for “days”, “weeks” or “months”. Model performance will also be assessed by plotting Kaplan-Meier survival curves for each of the three risk groups identified by the PiPs models (“days,” “weeks,” and “months+”). The model calibration will be assessed by comparing observed and predicted probabilities [37].

##### Comparison between PiPS-B model predictions and CES

To compare the accuracy of the model predictions and CES, the primary analysis will focus on the PiPS-B model. McNemar’s test will be used to compare the proportion of overall patient deaths predicted correctly by PIPS-B with the corresponding proportion predicted correctly by clinicians.

#### Secondary analyses

As part of the secondary analyses the PiPS models’ predictions for the two week and two month cut-off points will be combined to produce a categorical prediction of survival (“days,” “weeks,” or “months/years”) and will be compared with clinicians’ estimates and the corresponding observed values descriptively with respect to their accuracy. Linear weighted *k* will also be used to compare the performance of the clinicians with that of the models.

The PPI, PPS, PaP and FPN prognostic models will also be assessed as a part of the secondary analyses. The calibration of these prognostic models will be assessed using the calibration slope [37] based on a logistic model for binary outcomes and Cox model for survival outcomes [38]. Graphical comparisons of the observed and predicted risks for clinically relevant patient risk groups will also be made. Clinically relevant time points will be used for comparisons for survival outcomes. Model discrimination will be assessed using the C-statistic for binary outcomes and C-index for survival outcomes [39]. The performance measures estimated for the various models will be compared descriptively. The predictions made by these prognostic models will also be compared with the corresponding observed outcomes and clinician predictions (where available).

Bias due to missing data will be investigated and multiple imputation based on chained equations [40] will be used to impute missing predictor values if considered necessary.

### Interim results – Patient recruitment

**Fig. 1:**
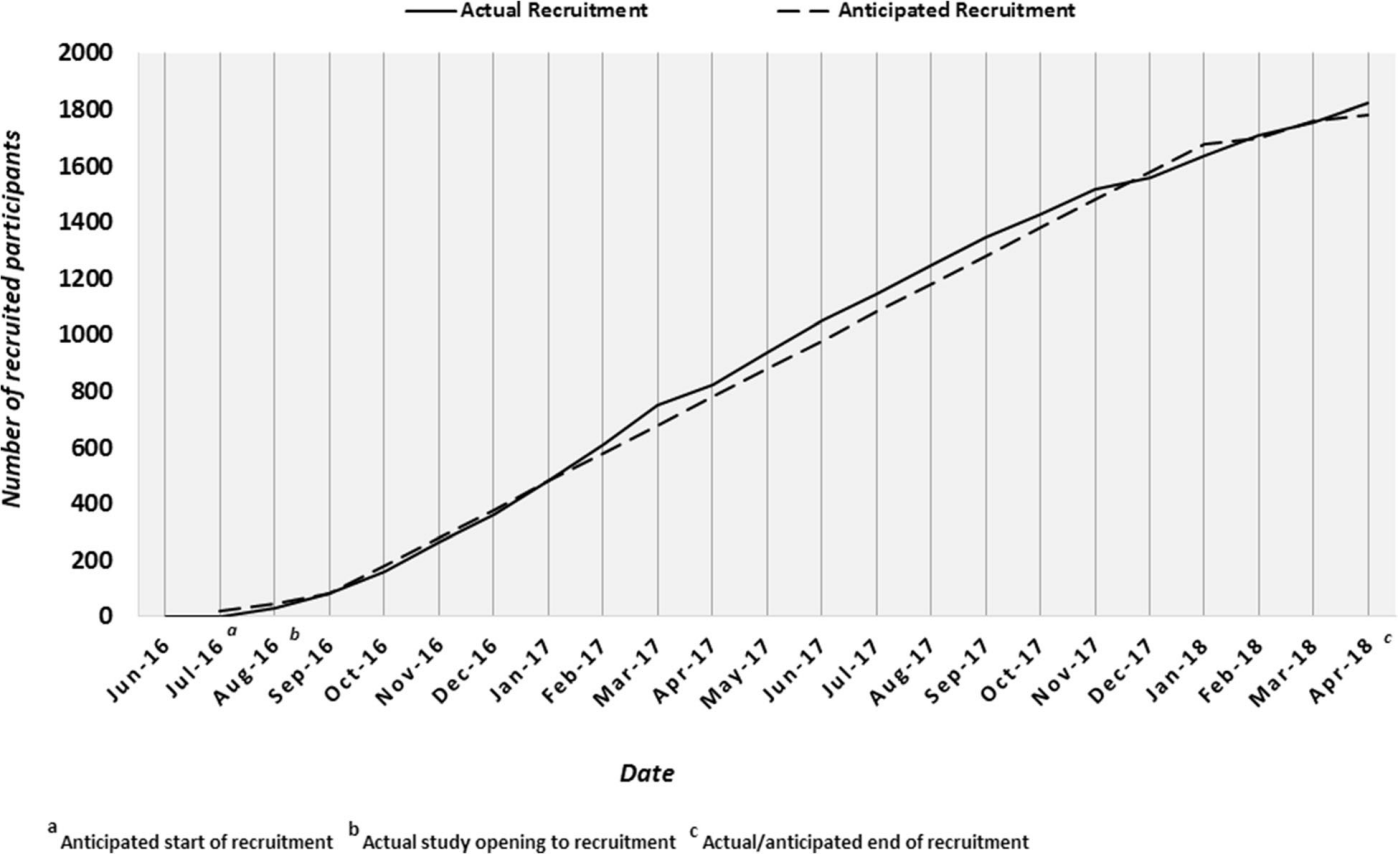
PiPS2 recruitment chart

Recruitment started in August 2016 and the required sample size was reached by the end April 2018 (see Fig. 1).

## Nested qualitative sub-study

Semi-structured, face to face interviews were conducted with a purposive sample of approximately 15 patients, 15 carers and 15 clinicians to determine the acceptability of using prognostic indicators, and barriers and facilitators to clinicians’ use. Data saturation will determine the final sample sizes, which may be larger or smaller than anticipated. To date, 28 patients, 19 carers and 32 clinicians were enrolled in the qualitative sub-study.

Interviews were audio recorded, and used iterative topic guides based on reviews of the literature, themes arising from preceding interviews, and the MORECare recommendations for conducting research at the end of life [41] The patient and carer interviews explored whether participants wish to know the patient’s prognosis, and if so whether they would have preferred clinicians’ estimates of survival or prognostic modelling. Those wishing to know their prognosis were asked how this information should have been presented to them. Interviews were of 30–60 min duration to ensure that participants were not overburdened.

The clinician interviews were interactive and explored the acceptability of PiPS, PPI, PPS, PAP and FPN. Participants were shown the prognostic models, and commented on their utility. Topic guides included questions about potential barriers and facilitators to using the models, and to discussing prognostic information with patients and carers. Interviews were of 60 min duration to allow enough time for in-depth discussion.

Interview data will be entered into NVivo 10.0 (https://www.qsrinternational.com/nvivo/) and analysed using the five stages of Framework Analysis [42]: familiarisation, developing a thematic framework, indexing, charting, and mapping and interpretation.

## Discussion

This study recruited the required sample size of 1778 patients across England and Wales by the end of April 2018. These data demonstrate that recruitment to a multi-centre prospective palliative care study is achievable. The results of this study, should permit a recommendation to be made about the accuracy of a variety of prognostic models, and whether or not the PiPS-B model is significantly better than a multi-professional MDT estimate of survival. In addition to this quantitative validation study, an embedded qualitative sub-study is also being undertaken. This is designed to assess the acceptability of the different prognostic models to patients, carers and clinicians and to make an assessment of the barriers to clinical use. We anticipate that the overall results of our research are expected to remain relevant and important to the needs of the NHS in the future as the number of patients with advanced cancer is anticipated to increase substantially over the next twenty years.

## Abbreviations

(e)CRF: (electronic) Case Report Form
CES: Clinicians’ Estimates of Survival
ECOG: Eastern Co-operative Oncology Group
FPN: Feliu Prognostic Nomogram
GP: General Practitioner
HSCIC: Health and Social Care Information Centre
IQ: Interquartile
KPS: Karnofsky Performance Scale
NHS: National Health Service
PaP: Palliative Prognostic score
PiPS: Prognosis in Palliative care Scales
PIS: Patient Information Sheet
PPI: Palliative Prognostic Index
PPS: Palliative Performance Scale
