# Correction to: The prognosis in palliative care study II (PiPS2): study protocol for a multi-centre, prospective, observational, cohort study

**DOI:** 10.1186/s12904-018-0373-6

**Published:** 2018-11-03

**Authors:** Anastasia K. Kalpakidou, Chris Todd, Vaughan Keeley, Jane Griffiths, Karen Spencer, Victoria Vickerstaff, Rumana Z. Omar, Patrick Stone

**Affiliations:** 1 Marie Curie Palliative Care Research Department, Division of Psychiatry, UCL, 6th Floor, Maple House, 149 Tottenham Court Road, London, W1T 7NF UK; 2 The School of Nursing, Midwifery and Social Work, University of Manchester, Manchester, M13 9PL UK; 3 Derby Teaching Hospitals NHS Foundation Trust, Derby, DE1 2QY UK; 4 Department of Statistical Science, UCL, London, WC1E 7HB UK

## Correction

After publication, the authors noticed some minor errors in “Nested qualitative sub-study” section, first paragraph of the section, page 7 of the published article. These may cause some confusion to the readers when the results of the qualitative sub-study are published [1].

Please see below for details (the new text has been added in bold and the incorrect text has been underlined.

“**It was anticipated that** semi-structured, face to face interviews were **would need to be** conducted with a purposive sample of approximately 15 patients, 15 carers and 15 clinicians to determine the acceptability of using prognostic indicators, and barriers and facilitators to clinicians’ use. **It was recognised that**, data saturation will **would** determine the final sample sizes, which may be larger or smaller than anticipated. To date, 28 patients, 19 carers and 32 clinicians were **have been** enrolled in the qualitative sub-study.”
